# Impact of COVID-19 on admission and in-hospital mortality of patients with acute myocardial infarction in Korea: An interrupted time series analysis

**DOI:** 10.1371/journal.pone.0316943

**Published:** 2025-02-21

**Authors:** Soo-Hee Hwang, Youngs Chang, Haibin Bai, Jieun Yun, Hyejin Lee, Jin Yong Lee

**Affiliations:** 1 HIRA Research Institute, Health Insurance Review & Assessment Service, Wonju-si, Gangwon-do, Republic of Korea; 2 Department of Preventive Medicine, Soonchunhyang University College of Medicine, Cheonan-si, Chungcheongnam-do, Republic of Korea; 3 Department of Health Policy and Management, Seoul National University College of Medicine, Seoul, Republic of Korea; 4 Department of Pharmaceutical Engineering, Cheongju University, Cheongju, Chungcheongbuk-do, Republic of Korea; 5 Department of Family Medicine, Seoul National University Bundang Hospital, Seongnam-si, Gyeonggi-do, Republic of Korea; 6 Public Healthcare Center, Seoul National University Hospital, Seoul, Republic of Korea; 7 Institute of Health Policy and Management, Seoul National University Medical Research Center, Seoul, Republic of Korea; Kyung Hee University School of Medicine, REPUBLIC OF KOREA

## Abstract

**Objectives:**

The purpose of this study is to investigate the impact of COVID-19 on admission and in-hospital mortality of patients with acute myocardial infarction (AMI).

**Methods:**

We constructed a dataset of monthly hospitalizations and mortality of inpatients with AMI from January 2017 to December 2021 utilizing the National Health Insurance Claims Data which covers nearly the entire population. Using an interrupted time series (ITS), we investigated how COVID-19 affected hospitalizations and in-hospital deaths of patients with AMI.

**Results:**

During the study period, the average age of patients with AMI was 65.2–65.8 years, and the ratio of men to women was higher, with 73.0–75.3% of patients being men and 24.7–27.0% being women. ITS analysis showed that admission rates of patients with AMI decreased one per 100,000 population due to COVID-19 (P<0.001). Reductions in admission rates were greatest among men, those aged 55 and older, and people with medical aid. COVID-19 did not affect inpatient mortality (p = 0.9608), but in-hospital mortality decreased from 12% to 7% in the medical aid group.

**Conclusion:**

Overall, we found that COVID-19 had an impact on admission rates of patients with AMI but did not have a significant impact on in-hospital mortality. However, we also found differential impacts by sex, age, and socioeconomic status, indicating some may be more vulnerable. This highlights the importance of identifying and supporting these vulnerable populations to prevent poorer health outcomes.

## Introduction

The first case of the Coronavirus disease 2019 (COVID-19) infection confirmed in December 2019 in China. As COVID-19 continues to spread worldwide, the World Health Organization (WHO) declared COVID-19 as a pandemic in March 2020. Globally reported death toll from COVID-19 between 2020 and 2021 was around 6 million, but the estimated cases of excess mortality were 18.2 million [1].

With the COVID-19 pandemic increasingly influencing public health, the quality of care in hospitals, especially general hospitals with relatively fewer beds [2], may have been negatively affected. As hospitals had become saturated with patients early in the pandemic, and access to healthcare services were limited due to social distancing, patients with emergency conditions [3] such as acute myocardial infarction (AMI) may not have received timely and appropriate treatment, which may have contributed to a spike in deaths [4–6]. Many studies have investigated that the COVID-19 pandemic caused a reduction in the number of patients who were hospitalized and visited emergency department for AMI [7–12].

However, these studies have several methodological issues. First, they did not use nationally representative data, which makes it difficult to measure overall effects. Second, since most of the studies were simple comparisons between before and after the pandemic, there was a limitation of applying time-series changes. Especially in an aging and rapidly changing society such as Korea, it is necessary to conduct interrupted time-series (ITS) analysis methods rather than simple comparisons between pre and post pandemic periods. Therefore, the purpose of this study is to investigate how much COVID-19 affected hospitalization and mortality of patients with AMI based upon ITS analysis and to identify what socioeconomic variables had a greater impact on their hospitalization and mortality.

## Methods

### Data source

We used the National Health Insurance Claims Data of the National Health Insurance Service (NHIS). It is a national healthcare data of almost the whole population of Korea, including beneficiaries of National Health Insurance (NHI) and Medical Assistance (MA). In addition to this, this claims data indicates diagnoses, encoded with the Korean Standard Classification of Diseases (KCD) codes based on the International Classification of Diseases (ICD), treatment, prescription, and provider data [13, 14].

We also used annual statistics on NHI and MA beneficiaries, whose sources came from the Health Insurance Review and Assessment Service (HIRA) and the NHIS. These statistics include the number of the NHI and the MA beneficiaries by age and sex as of the end of the year [15]. The NHIS completely anonymized the personal identification information of the data before it was provided to the researchers. The date of access was February 1^st^, 2024.

### Study population and design

We identified patients aged 20 years or older who were admitted to hospitals from January 1, 2017 to December 31, 2021 for acute non-elective (urgent) care, diagnosed with AMI (KCD-8: I21 and I22) as a principal disease. We delved into the study patients in reflecting all admissions ranging from day cases to the transfer cases to another facilities for the acute care. Apart from age, the study subjects can be leveraged as the denominator in AMI 30-day mortality with unlinked data. Basically, this indicator is used by the OECD to measure quality and outcomes in the acute care domain [16].

We performed an interrupted time series (ITS) analysis using segmented regression with a single interruption to assess the impact of the COVID-19 pandemic on the monthly AMI healthcare utilization and outcomes (hospitalization rate and 30-days mortality). The ITS studies are frequently used to evaluate the impact of interventions or exposures that occur at a specific point in time. In particular, in real-world situations such as natural disasters or pandemics, the ITS design can be considered one of the most appropriate alternative experimental designs to evaluate these effects [17, 18].

The single point intervention was set at February 2020, given that the first confirmed case in Korea occurred on 20 January 2020. The Korean government raised the COVID-19 alert level to the highest (level 4) on 23 February 2020 (Shim et al., 2020). Based on this single point in time, the pre-COVID-19 period for 37 months from January 2017 to January 2020 was compared with the post-COVID-19 period during 23 months from February 2020 to December 2021.

### Statistical analysis

The monthly acute rates of AMI hospitalization per 100,000 and AMI 30-day mortality rates in hospital (crude rate by age group, sex, type of insurance, and Charlson Comorbidity Index group) were used as outcome variables in the ITS analysis to study the impact of the pandemic. To test whether there were differences in hospitalization and mortality within demographic groups, we also calculated the AMI hospitalization and mortality rates by age groups (20–44, 45–54, 55–64, 65–74, 75–84, and over 85 years), sex, and type of insurance—NHI and MA program.

ITS analysis was performed to determine the significance of changes in the indicators for the overall trend (time), the onset of the pandemic on February 2020 (intervention), and trends after the outbreak of the pandemic (time after intervention). The equation for the segmented regression model is as follows: Yt = β0 + β1 × Time + β2 × Intervention + β3 × Time after intervention t + εt. Where β0 is the baseline level (intercept) of the outcome variable, β1 is the slope of the outcome variable before the outbreak (the pre-existing trend), β2 is the change in the value of the outcome variable immediately after the outbreak (compared to the counterfactual). β3 represents the difference between the slopes of the outcome variable before and after the pandemic. For interpretation, we presented absolute and relative changes using the predicted and observed values at the time of the COVID-19 pandemic and at the end of the observation period. The presence of first-order autocorrelation was tested using the Durbin-Watson statistic.

All statistical analyses were performed using SAS Enterprise Guide 7.4 (SAS Institute Inc., Cary, North Carolina, USA) with a two-sided significance level of 0.05.

### Ethics statement

This study was reviewed and approved by the institutional review board (IRB) of Seoul National University Bundang Hospital (IRB No. X-2304-820-902). Informed consent was waived as we used anonymized data.

## Results

### General characteristics of patients with AMI in Korea (2017–2021)

Table 1 shows general characteristics of the study populations. It shows that the number of hospitalized patients with AMI increased from 21,973 in 2017 to 26,349 in 2021. During the study period, the average age of patients with AMI was 65.2–65.8 years, and the ratio of men to women was higher, with 73.0–75.3% of patients being men and 24.7–27.0% being women.

**Table 1:** General characteristics of study subjects.

	2017		2018		2019		2020		2021		Total
Total no. of hospitalization	21,973		25,701		26,375		25,303		26,349		125,701
Age, mean years (SD)	65.2	13.4	65.6	13.3	65.6	13.4	65.6	13.3	65.8	13.3	
20–44	1,298	5.9%	1,476	5.7%	1,496	5.7%	1,339	5.3%	1,483	5.6%	7,092
45–54	3,775	17.2%	4,102	16.0%	4,293	16.3%	4,099	16.2%	4,096	15.6%	20,365
55–64	5,602	25.5%	6,582	25.6%	6,881	26.1%	6,743	26.7%	6,957	26.4%	32,765
65–74	4,920	22.4%	5,861	22.8%	5,895	22.4%	5,797	22.9%	6,300	23.9%	28,773
75–84	4,862	22.1%	5,827	22.7%	5,791	22.0%	5,347	21.1%	5,361	20.4%	27,188
85-	1,516	6.9%	1,853	7.2%	2,019	7.7%	1,978	7.8%	2,152	8.2%	9,518
Sex											
Men	16,040	73.0%	18,798	73.1%	19,548	74.1%	18,971	75.0%	19,836	75.3%	93,193
Women	5,933	27.0%	6,903	26.9%	6,827	25.9%	6,332	25.0%	6,513	24.7%	32,508
Type of Insurance											
NHIS	20,374	92.7%	23,680	92.1%	24,322	92.2%	23,389	92.4%	24,301	92.2%	116,066
MA	1,599	7.3%	2,021	7.9%	2,053	7.8%	1,914	7.6%	2,048	7.8%	9,635
CCI											
0	9,447	43.0%	10,996	42.8%	11,255	42.7%	11,148	44.1%	11,432	43.4%	54,278
1–2	7,312	33.3%	8,541	33.2%	8,563	32.5%	8,084	32.0%	8,359	31.7%	40,859
3-	5,214	23.7%	6,164	24.0%	6,557	24.9%	6,071	24.0%	6,558	24.9%	30,564
COVID+	0	0.0	0	0.0	0	0.0	1	0.0	3	0.0	4

Note: COVID+ indicates cases of COVID-19 in the secondary diagnosis.

Specifically, the number and proportion of AMI hospitalization cases were highest in the over 55 age group, while the proportion of AMI admission cases was less than 6% in the 20–44 years age group. Additionally, men outnumbered women by a ratio of three to one. About 92% of AMI admission cases were covered by the NHI, and approximately 8% came from the MA recipients. A CCI score of zero was the most common, followed by one to two, with more than three being the least common.

### Changes in hospitalization rates for patients with AMI and the impact of COVID-19 pandemic

**Fig 1:**
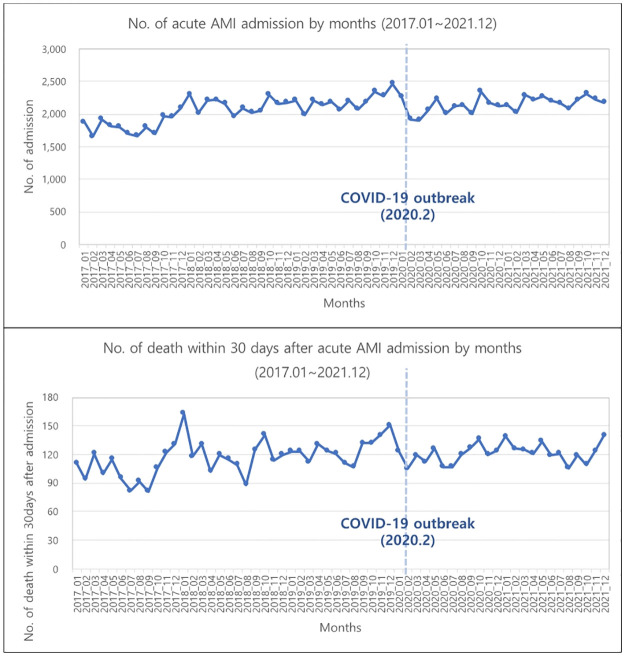
Number of acute myocardial infarction admission and deaths within 30 days after acute myocardial infarction admission by months.

**Fig 2:**
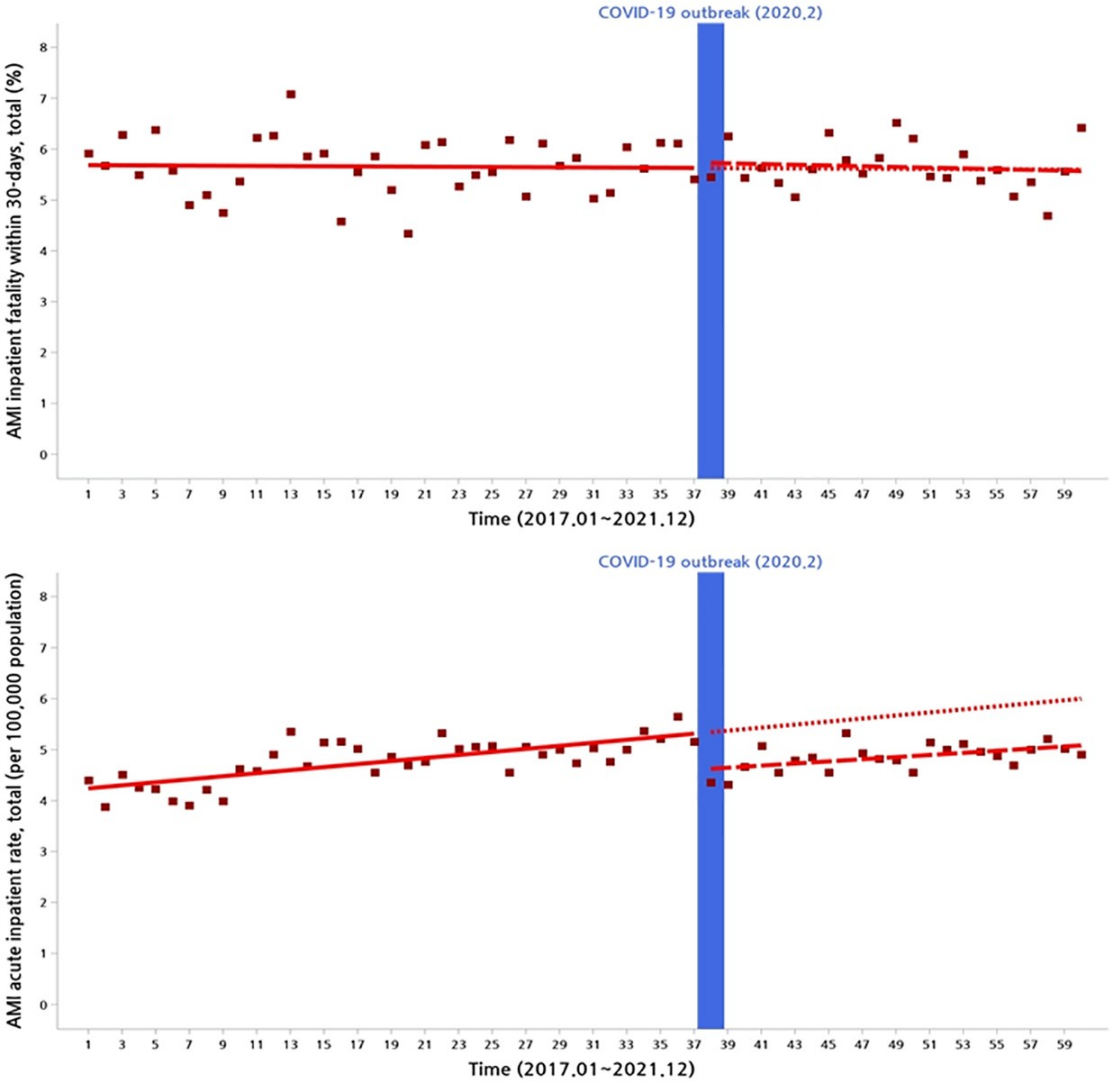
Acute myocardial infarction inpatient rate and in-hospital mortality within 30 days.

Fig 1 is a scatter plot that indicates the monthly number of AMI, which had fluctuated up and down throughout the period. The number of AMI admission patients ranged between 1,600 and 2,500. The figure stood at 1,922 right before the COVID-19 pandemic. AMI inpatient rates showed a different pattern in Fig 2. In this graph, around 5 per 100,000 was hospitalized for acute AMI during the pre-pandemic period. However, this figure dropped to about 4 per 100,000 after the outbreak of the COVID-19 pandemic. This indicates that the COVID-19 had an outstanding impact on the number of the inpatients with AMI. Table 2 shows a slope for the time and intervention at the time of the COVID-19 pandemic for AMI admission rates. Estimates calculated by ITS in AMI admission rate are presented in S1 Table.

**Table 2:** Slope for the time and intervention, absolute and relative changes between predicted and actual values at the time of the COVID-19 pandemic and the end of the observation: Acute myocardial infarction admission rate.

		Time			Intervention			Time after Intervention			Change at Intervention (February 2019)		Change at December 2021	
		Estimate	Std. Error	P value	Estimate	Std. Error	P value	Estimate	Std. Error	P value	Absolute	Relative	Absolute	Relative
Total		0.0298	0.004293	<.0001	-0.7092	0.15	<.0001	-0.00897	0.009758	0.3617	-1	-13	-1	-15
Age group	20–44	0.004192	0.001058	0.0002	-0.1395	0.037	0.0004	0.002113	0.002405	0.3834	0	-19	0	-11
	45–54	0.0173	0.004418	0.0003	-0.3434	0.1544	0.0302	-0.0106	0.01	0.2966	0	-9	-1	-13
	55–64	0.0393	0.00659	<.0001	-0.9003	0.2303	0.0003	-0.0137	0.015	0.3655	-1	-12.3692	-1	-15
	65–74	0.0351	0.0171	0.045	-1.6293	0.5749	0.0064	-0.0126	0.0389	0.7465	-2	-14.4706	-2	-16
	75–84	0.0772	0.0308	0.0152	-3.3872	1.0164	0.0015	-0.0393	0.0702	0.5778	-3	-18.1137	-4	-20.8158
	85+	0.0961	0.0295	0.002	-3.9062	1.0739	0.0006	-0.0584	0.0683	0.3966	-4	-16.9028	-5	-20.5297
Sex	Men	0.0477	0.006494	<.0001	-0.9988	0.227	<.0001	-0.013	0.0148	0.3815	-1	-13	-1	-14
	Women	0.0118	0.002796	<.0001	-0.4167	0.0977	<.0001	-0.00478	0.006357	0.4548	0	-16	-1	-18
Insurance	NHI	0.0275	0.00398	<.0001	-0.6497	0.1391	<.0001	-0.00814	0.009047	0.3722	-1	-13	-1	-15
	MA	0.1003	0.1003	0.1003	-2.7246	0.7616	0.0007	-0.0381	0.0495	0.4448	-3	-19	-4	-22

The AMI acute inpatient rate by sex is shown in S1 Fig. AMI inpatient rate per 100,000 population demonstrates the clear impact of COVID-19 on both men and women. For instance, following the COVID-19 pandemic, the AMI acute inpatient rate for men dropped from 7 to 6 per 100,000, and the rate for women decreased from 2.5 to 2 per 100,000.

S2 Fig displays the AMI acute inpatient rates by age group. The crude rate per 100,000 differed across age groups. Among those aged 55–64, 65–74, 75–84, and 85+, the rate showed the impact of COVID-19 pandemic, with the differences becoming more conspicuous with increasing age. In contrast, the rate in the 20–44 age groups remained consistent before and after the outbreak of the pandemic.

AMI acute inpatient rate, analyzed by insurance types, is presented in S3 Fig. The COVID-19 pandemic affected the AMI acute inpatient rate per 100,000 for both the NHI and MA beneficiaries. Both groups experienced a decrease in the crude rate after the COVID-19 pandemic with a more significant impact observed in the MA group. Specifically, the AMI acute inpatient rate in the MA group decreased from 14 per 100,000 before the outbreak to 11 per 100,000 after the outbreak.

### Changes in in-hospital mortality for patients with AMI and the impact of COVID-19 pandemic

The monthly number of deaths within 30 days after AMI admission is shown in a scatter plot in Fig 1, which indicates a constant fluctuation for a certain period of time. The number of deaths within 30 days after AMI admission ranged from 82 to 163, with 105 before the COVID-19 pandemic.

Although AMI admission rate showed a decreased pattern, there was no apparent impact of the COVID-19 pandemic on the AMI in-hospital mortality within 30 days. Table 3 shows the slope for the time and intervention at the time of the COVID-19 pandemic for AMI in-hospital mortality. Estimates calculated by ITS in AMI in-hospital mortality are presented in S2 Table.

**Table 3:** Slope for the time and intervention, absolute and relative changes between predicted and actual values at the time of the COVID-19 pandemic and the end of the observation: Acute myocardial infarction inpatient mortality within 30 days.

		Time			Intervention			Time after Intervention			Change at Intervention (February 2019)		Change at December 2021	
		Estimate	Std. Error	P value	Estimate	Std. Error	P value	Estimate	Std. Error	P value	Absolute	Relative	Absolute	Relative
Total		-0.0015	0.008255	0.8566	0.1049	0.2885	0.7174	-0.00568	0.0188	0.7634	0	2	0	0
Age group	20–44	0.0223	0.0142	0.1224	-0.9161	0.4975	0.0709	0.0150	0.0324	0.6447	-1	-52	-1	-26
	45–54	-0.0166	0.0121	0.1735	0.2356	0.4218	0.5786	0.0190	0.0274	0.4906	0	17	1	57
	55–64	-0.00666	0.0104	0.5262	0.8125	0.3652	0.0301	-0.0316	0.0238	0.1889	1	32	0	4
	65–74	-0.0239	0.0156	0.1306	0.2713	0.5448	0.6205	0.001926	0.0354	0.9569	0	6	0	7
	75–84	0.0214	0.0208	0.3084	0.0987	0.7282	0.8926	-0.0308	0.0474	0.5184	0	1	-1	-6
	85+	-0.0115	0.0348	0.7431	-1.5179	1.2673	0.2362	0.0827	0.0797	0.3039	-1	-8	0	2
Sex	Men	0.004290	0.005664	0.4520	-0.0710	0.2168	0.7445	-0.00858	0.0133	0.5216	0	-2	1	12
	Women	-0.0112	0.0184	0.5469	1.0180	0.6439	0.1195	-0.0113	0.0419	0.7880	0	-6	1	9
Insurance	NHI	-0.00714	0.005871	0.2289	0.2896	0.2202	0.1938	-0.00218	0.0136	0.8734	0	5	0	5
	MA	0.0606	0.0291	0.0420	-0.732	1.1151	0.5143	-0.0915	0.0685	0.1868	-1	-8	-3	-26

AMI in-hospital mortality within 30 days is presented in S1 Fig. The data shows different mortality between men and women. After the COVID-19 pandemic, the mortality of women increased from 9% to 10%. In contrast, the mortality of men showed no difference before and after the pandemic.

S2 Fig demonstrates AMI in-hospital mortality within 30 days by age group. The pattern of the AMI in-hospital mortality within 30 days by age group was different from the AMI inpatient rate. In 20–44, and 85+age groups, AMI in-hospital mortality within 30 days decreased immediately after the COVID-19 pandemic but increased over time.

AMI in-hospital mortality within 30 days is presented by insurance types in S3 Fig. Both the NHI and the MA groups exhibited different patterns in the AMI in-hospital mortality within 30 days. While the NHI group showed no significant difference, the MA group experienced a clear impact with the AMI in-hospital mortality within 30 days decreasing from 12% to 7%.

## Discussion

The key findings from this study show that the COVID-19 reduced admission rates for patients with AMI but did not have a significant impact on in-hospital mortality. However, we also found differential impacts by sex, age, and socioeconomic status. In particular, the MA recipients experienced an apparent reduction in both admission rates and in-hospital mortality compared to the NHI beneficiaries.

This study has the following advantages. First, we used national level data to measure the impact of COVID-19 on AMI acute admission rate and in-hospital mortality. Second, instead of a simple before and after comparison, we conducted ITS analysis. It is a well-known method for evaluating the impact of population-level interventions at a specific point in time [19]. This method is particularly useful in a fast-changing society, such as Korea, which is also experiencing an aging population.

It has been known that the reduction in hospitalization of patients with AMI might have led to an increased mortality due to delayed time of appropriate treatment [20–28]. As mentioned earlier, this study shows that AMI acute inpatient rate decreased in older age groups, over 55 years, and the MA group went through a more reduction in AMI acute inpatient rate than the NHI group did. Since the COVID-19 pandemic, there has been a long-term trend of increased hospitalization rates. Several factors are thought to be responsible for the decrease in the proportion of AMI inpatient rate. The implementation of social distancing and quarantine measures against the COVID-19 pandemic could potentially affect the overall treatment of AMI. Such policies were aggressively pushed ahead by the government so that individuals might stay away from infections. Furthermore, limited medical resources and lack of quarantine facilities may deter patients from seeking care in the emergency rooms.

However, the trend of AMI in-hospital mortality within 30 days showed no difference before and after the outbreak, except among women and the MA group. This finding is in stark contrast to the previous studies which showed higher in-hospital mortality rates. The lack of change in in-hospital mortality can be interpreted as a sign that the quality of hospital admission has been maintained. The trend of AMI in-hospital mortality within 30 days after the COVID-19 pandemic increased only in women, while the trend decreased among the MA group. Women saw a less reduction in AMI acute inpatient rates but showed higher in-hospital mortality rates than men did. This could be interpreted that women had a longer delay of time for the treatment since they have the longest time from symptom onset to emergency departments, showing the lowest rate of emergency room visits [11]. The observed decrease in in-hospital mortality rates for AMI among the MA group during the COVID-19 pandemic may be linked to a reduction in AMI hospitalizations, particularly noted in the early stages of the pandemic. Gluckman et al. reported that AMI hospitalizations decreased at a rate of 19.0 cases per week over a 5-week period during the early phase of the COVID-19 pandemic [29]. This decline allowed healthcare facilities to reallocate their resources and deliver more effective care to those who did seek treatment. Furthermore, the Centers for Disease Control and Prevention (CDC) reported a 42% decrease in emergency department visits during the pandemic’s initial phase, which likely contributed to an environment with enhanced medical capacity [30]. However, it is also important to consider that severely ill MA patients may have faced greater challenges in accessing emergency departments compared to NHI patients, potentially resulting in higher rates of out-of-hospital deaths and a relative decrease in in-hospital mortality.

Although we first investigated the impact of COVID-19 pandemic on AMI admission and in-hospital mortality by socioeconomic position, there are several limitations to this study. First, this study did not account for variables that may influence AMI, particularly in relation to COVID-19 and other relevant factors. Utilizing ITS analysis. Potential biases may arise from various interventions implemented in response to COVID-19, including quarantine measures, lockdowns, alterations in healthcare service delivery, and seasonal variations. Nevertheless, the analysis stratified the population by age, sex, and insurance type to examine the differential impact of COVID-19 based on demographic characteristics. Future research is anticipated to address additional variables that may introduce bias, which were not considered in the current study. Second, we found that fewer patients were hospitalized, and mortality rates were not different among the inpatients. We may possibly assume that people who died in the community without being hospitalized were not included in our analysis. Therefore, additional deaths may have occurred. In this study, the MA patients had fewer hospitalizations and lower in-hospital mortality rates. It can be hypothesized that there may have been some deaths in the MA group that were not captured in the hospitalization data. However, the findings of this research are not consistent with the earlier studies conducted in Korea. Further analysis of matching with mortality data from Statistics Korea is needed to confirm this. Third, our study is based on the assumption that only the COVID-19 pandemic affected AMI inpatient rate and in-hospital mortality. We cannot rule out the possibility that factors other than the outbreak may have influenced the AMI admission and in-hospital mortality. Fourth, the data we used is from the national health insurance claim data. While this data has the advantage of including a larger number of people, it is limited in that it cannot provide detailed hospital medical records, as the claim data is not a medical record review. Fifth, there is a methodological issue with interrupted time series analysis [19]. One limitation is the lack of control for time-varying confounders. Therefore, any interpretations regarding the effect of COVID-19 should be made with vigilance. Nevertheless, interrupted time series analysis is one of the strongest evaluative methods when randomization is not feasible, as it allows an assessment of the longitudinal impact of an intervention.

AMI is a potentially fatal condition that requires urgent treatment as soon as the diagnosis is made. In terms of future policy recommendations, it would be ideal for the healthcare system to maintain appropriate accessibility to hospital without reference to socioeconomic position.

In conclusion, this study found that the COVID-19 pandemic led to a decrease in AMI admission rates across age group, sex, and socioeconomic position. Additionally, AMI in-hospital mortality stayed unchanged except among women and the MA patients. It is important to note that different populations may experience different outcomes during a health crisis, indicating that some populations may be more vulnerable. This highlights the importance of identifying and supporting those vulnerable populations to prevent poorer health outcomes. As new pandemics may arise and escalate in the future, it is crucial to strengthen public healthcare system to ensure equitable access and support for all communities.

## Supporting information

S1 Fig AMI admission rate and in-hospital mortality within 30 days by sex. (TIFF)

S2 Fig AMI admission rate and in-hospital mortality within 30 days by age group. (TIFF)

S3 Fig AMI admission rate and in-hospital mortality within 30 days by insurance type. (TIFF)

S1 Table Estimate by variables: Acute myocardial infarction admission rate. (DOCX)

S2 Table Estimate by variables: Acute myocardial infarction in-hospital mortality within 30 days. (DOCX)
