# Supplementary figures and images for: Impact of COVID-19 on admission and in-hospital mortality of patients with acute myocardial infarction in Korea: An interrupted time series analysis
Source: PLoS One. 2025 Feb 21;20(2):e0316943. doi: 10.1371/journal.pone.0316943 (PMC11844859; doi:10.1371/journal.pone.0316943)

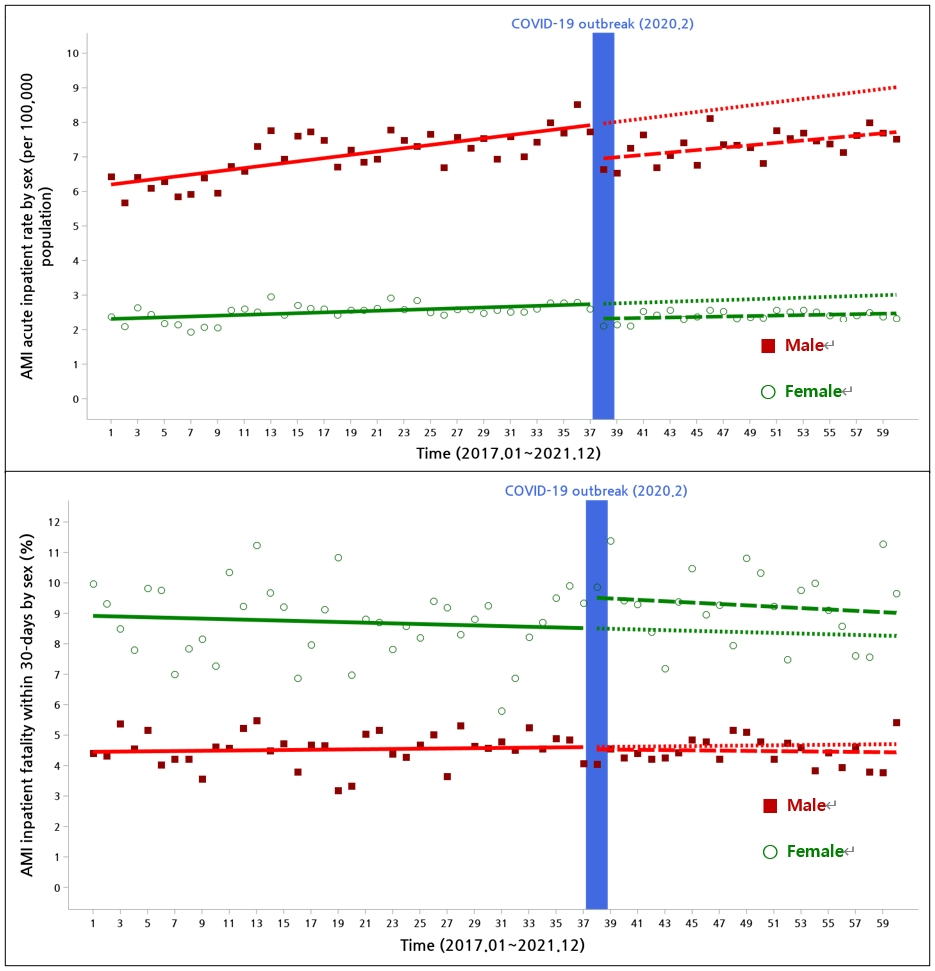

Supplement: S1 Fig — (TIFF) [file pone.0316943.s001.tiff]

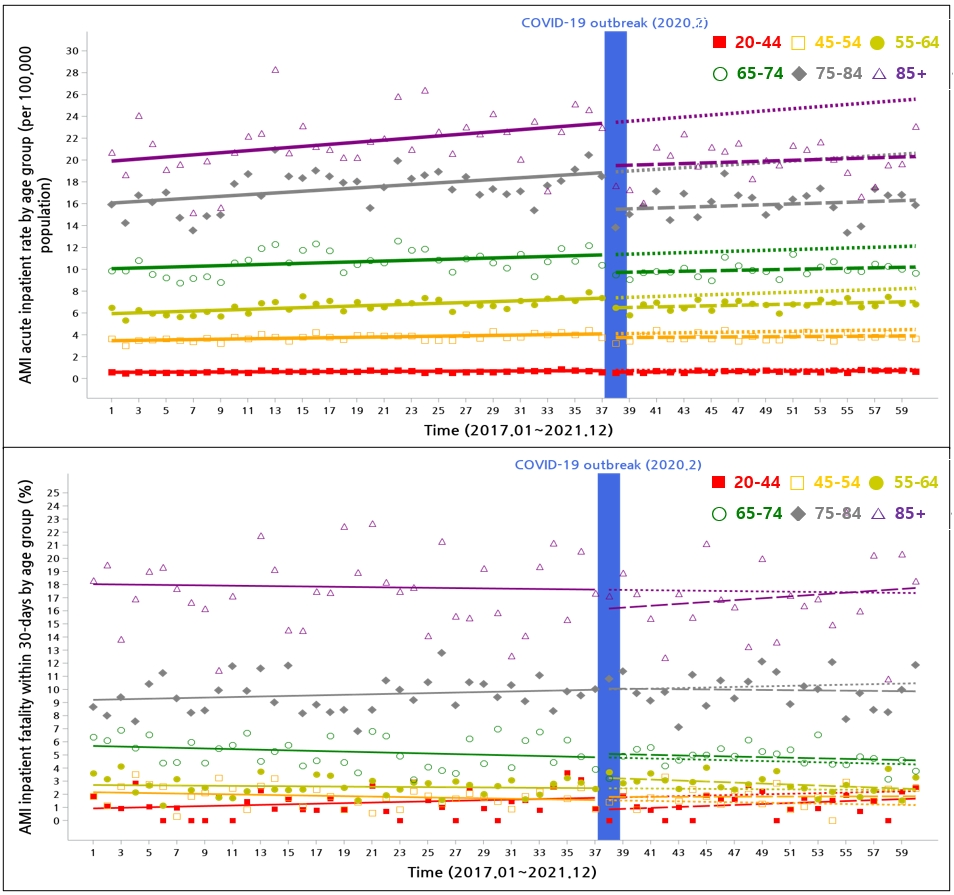

Supplement: S2 Fig — (TIFF) [file pone.0316943.s002.tiff]

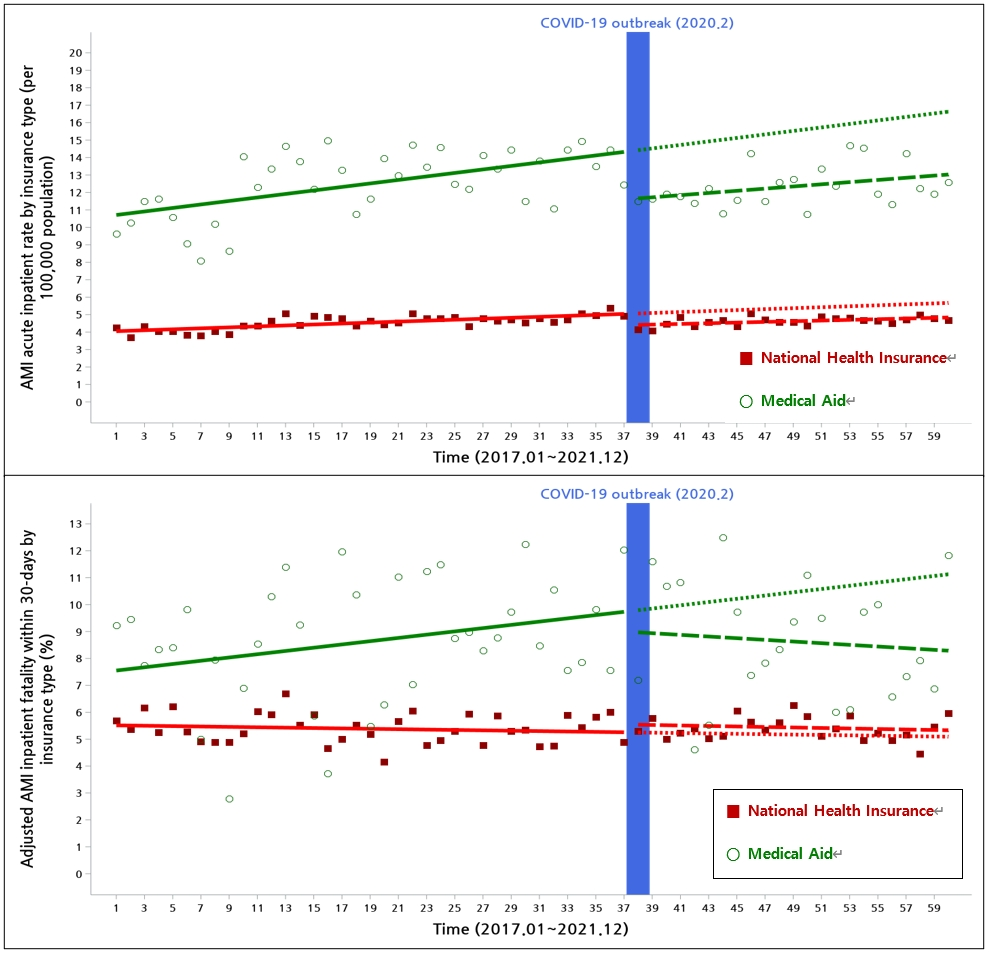

Supplement: S3 Fig — (TIFF) [file pone.0316943.s003.tiff]
