# Supplementary material for: Impact of COVID-19 on admission and in-hospital mortality of patients with acute myocardial infarction in Korea: An interrupted time series analysis
Source: PLoS One. 2025 Feb 21;20(2):e0316943. doi: 10.1371/journal.pone.0316943 (PMC11844859; doi:10.1371/journal.pone.0316943)
Supplement: S1 Table — (DOCX) [file pone.0316943.s004.docx]

S1 Table. Estimate by variables: acute myocardial infarction admission rate.

|  |  | Time | | | Intervention | | | Time after Intervention | | |
| --- | --- | --- | --- | --- | --- | --- | --- | --- | --- | --- |
|  |  | Estimate | Std. Error | p-value | Estimate | Std. Error | p-value | Estimate | Std. Error | p-value |
| Total |  | 0.0298 | 0.004293 | <.0001 | -0.7092 | 0.15 | <.0001 | -0.008974 | 0.009758 | 0.3617 |
| Age group | 20-44 | 0.004192 | 0.001058 | 0.0002 | -0.1395 | 0.037 | 0.0004 | 0.002113 | 0.002405 | 0.3834 |
|  | 45-54 | 0.0173 | 0.004418 | 0.0003 | -0.3434 | 0.1544 | 0.0302 | -0.0106 | 0.01 | 0.2966 |
|  | 55-64 | 0.0393 | 0.00659 | <.0001 | -0.9003 | 0.2303 | 0.0003 | -0.0137 | 0.015 | 0.3655 |
|  | 65-74 | 0.0351 | 0.0171 | 0.045 | -1.6293 | 0.5749 | 0.0064 | -0.0126 | 0.0389 | 0.7465 |
|  | 75-84 | 0.0772 | 0.0308 | 0.0152 | -3.3872 | 1.0164 | 0.0015 | -0.0393 | 0.0702 | 0.5778 |
|  | 85+ | 0.0961 | 0.0295 | 0.002 | -3.9062 | 1.0739 | 0.0006 | -0.0584 | 0.0683 | 0.3966 |
| Sex | Men | 0.0477 | 0.006494 | <.0001 | -0.9988 | 0.227 | <.0001 | -0.013 | 0.0148 | 0.3815 |
|  | Women | 0.0118 | 0.002796 | <.0001 | -0.4167 | 0.0977 | <.0001 | -0.004784 | 0.006357 | 0.4548 |
| Insurance | NHI | 0.0275 | 0.00398 | <.0001 | -0.6497 | 0.1391 | <.0001 | -0.008139 | 0.009047 | 0.3722 |
|  | MA | 0.1003 | 0.1003 | 0.1003 | -2.7246 | 0.7616 | 0.0007 | -0.0381 | 0.0495 | 0.4448 |
