# Supplementary material for: Impact of COVID-19 on admission and in-hospital mortality of patients with acute myocardial infarction in Korea: An interrupted time series analysis
Source: PLoS One. 2025 Feb 21;20(2):e0316943. doi: 10.1371/journal.pone.0316943 (PMC11844859; doi:10.1371/journal.pone.0316943)
Supplement: S2 Table — (DOCX) [file pone.0316943.s005.docx]

S2 Table. Estimate by variables: acute myocardial infarction in-hospital mortality within 30 days.

|  |  | Time | | | Intervention | | | Time after Intervention | | |
| --- | --- | --- | --- | --- | --- | --- | --- | --- | --- | --- |
|  |  | Estimate | Std. Error | p-value | Estimate | Std. Error | p-value | Estimate | Std. Error | p-value |
| Total |  | -0.001498 | 0.008255 | 0.8566 | 0.1049 | 0.2885 | 0.7174 | -0.005676 | 0.0188 | 0.7634 |
| Age group | 20-44 | 0.0223 | 0.0142 | 0.1224 | -0.9161 | 0.4975 | 0.0709 | 0.0150 | 0.0324 | 0.6447 |
|  | 45-54 | -0.0166 | 0.0121 | 0.1735 | 0.2356 | 0.4218 | 0.5786 | 0.0190 | 0.0274 | 0.4906 |
|  | 55-64 | -0.006664 | 0.0104 | 0.5262 | 0.8125 | 0.3652 | 0.0301 | -0.0316 | 0.0238 | 0.1889 |
|  | 65-74 | -0.0239 | 0.0156 | 0.1306 | 0.2713 | 0.5448 | 0.6205 | 0.001926 | 0.0354 | 0.9569 |
|  | 75-84 | 0.0214 | 0.0208 | 0.3084 | 0.0987 | 0.7282 | 0.8926 | -0.0308 | 0.0474 | 0.5184 |
|  | 85+ | -0.0115 | 0.0348 | 0.7431 | -1.5179 | 1.2673 | 0.2362 | 0.0827 | 0.0797 | 0.3039 |
| Sex | Men | 0.00429 | 0.005664 | 0.4520 | -0.0710 | 0.2168 | 0.7445 | -0.008583 | 0.0133 | 0.5216 |
|  | Women | -0.0112 | 0.0184 | 0.5469 | 1.0180 | 0.6439 | 0.1195 | -0.0113 | 0.0419 | 0.7880 |
| Insurance | NHI | -0.007143 | 0.005871 | 0.2289 | 0.2896 | 0.2202 | 0.1938 | -0.00218 | 0.0136 | 0.8734 |
|  | MA | 0.0606 | 0.0291 | 0.0420 | -0.732 | 1.1151 | 0.5143 | -0.0915 | 0.0685 | 0.1868 |
